# Delivery of Telehealth Complementary and Integrated Health Interventions Improves Mental Health and Overall Wellness to Broaden Veterans' Access to Care

**DOI:** 10.1089/jicm.2022.0614

**Published:** 2023-02-08

**Authors:** Nithya Ganesh, Shengnan Sun, Ann Feder, Hanga Galfalvy, Rachel Yehuda, Lauren Byma, Fatemeh Haghighi

**Affiliations:** ^1^ James J. Peters VA Medical Center, Bronx, NY, USA.; ^2^ Icahn School of Medicine at Mount Sinai, New York, NY, USA.; ^3^ Columbia University Medical Center, New York, NY, USA.

**Keywords:** suicide prevention, complementary integrated health, telehealth, depression, PTSD, pain

## Abstract

**Background:**

Complementary and integrative health (CIH) interventions show promise in improving overall wellness and engaging Veterans at risk of suicide.

**Methods:**

An intensive 4-week telehealth CIH intervention programming was delivered motivated by the COVID-19 pandemic, and outcomes were measured pre–post program completion.

**Results:**

With 93% program completion (121 Veterans), significant reduction in depression and post-traumatic stress disorder symptoms were observed pre–post telehealth CIH programing, but not in sleep quality. Improvements in pain symptoms, and stress management skills were observed in Veterans at risk of suicide.

**Discussion:**

Telehealth CIH interventions show promise in improving mental health symptoms among at-risk Veterans, with great potential to broaden access to care toward suicide prevention.

## Introduction

In the United States, suicide is the 12th leading cause of death, with Veterans accounting for ∼15% of these suicide deaths. Novel public health approaches are needed to address this alarming rates of Veteran suicides. Complementary and Integrative Health (CIH) interventions offer promising therapeutic potential for improving overall wellness in Veterans at risk for suicide, and in engaging Veterans to receive mental health care who typically do not engage, due to stigma or other barriers.^1,2^ In previous study, the authors have shown that in-person programming using intensive multimodal CIH interventions resulted in significant reduction in suicidal ideation and associated mental health risk factors, and had high Veteran engagement.^3^ With the emergence of the COVID-19 pandemic, the in-person multimodal CIH intervention was pivoted to a virtual delivery of care, and in this program evaluation study the authors set out to see whether the virtual mode of delivery was similarly effective.

## Methods

### Study design

The telehealth CIH interventions were administered as part of the Resilience and Wellness Center (RWC) located at the James J Peters VA Medical Center. The RWC program model is cohort based, consisting of ∼6 Veterans per cohort. As previously described,^3^ inclusion is transdiagnostic, targeting at-risk vulnerable Veterans who are isolated and experiencing ongoing environmental stressors, and who would like to learn new coping strategies and attempt to make significant lifestyle changes.

Since prior suicide attempt is a prominent risk factor for future suicidal behavior, in line with the authors' prior in-person program evaluation,^3^ for this virtual program evaluation study, participants' engagement and treatment outcomes pre- and post-telehealth RWC program completion were also investigated by history of suicide ideation and/or attempt and no history of ideation or attempt separately, because these two groups potentially differ in their risk severity. Veterans utilized either Veteran Video Connect (VVC) or Zoom platforms to attend the virtual classes, which were 2 h daily, 5 days per week, for 4 weeks.

The CIH interventions included mindfulness/meditation, yoga, horticultural therapy, nutrition/cooking, narrative therapy, sleep hygiene, exercise, and spirituality based on the authors' previously reported in-person CIH interventions^3^ (see Supplementary Table S1 for representative weekly schedule). To assess the effectiveness of the virtual delivery of the CIH interventions, an assessment battery was administered online through Qualtrics XM, a secure modifiable customer experience management software, at baseline and after program completion.

Veterans self-reported on their depressive symptoms, stress, pain, and sleep quality using validated instruments described previously.^3^ These assessments included the Patient Health Questionnaire (PHQ-9),^4^ the PTSD Checklist (PCL-5),^5^ Measures of Current Status (MoCS),^6^ Defense Veteran Pain Rating Scale (DVPRS),^7^ and the Pittsburgh Sleep Quality Index.^8^

### Statistical and data analysis

Analyses were performed on pre–post score differences for each of these assessments, with scores initially plotted by group to inspect for outliers. Outliers, defined as values outside 1.5 times the interquartile range above the third and below the first quartiles, were winsorized—censored to the nearest nonoutlier value to avoid deleting several observations from the analysis.^9^ Using the pre–post score differences, the authors did a one-sided *t* test to test if the assessments score improved after RWC program for the two groups separately and all participant together. Benjamini–Hochberg procedure was applied to control for false discovery rate with threshold set to be 0.05. Cohen's *d* was used to measure RWC treatment effects.

### Ethical and exemption statement

The RWC program evaluation was reviewed and determined by the Institutional Review Board and the Quality Improvement Executive Committee of the VA hospital to be exempt from IRB review, and it was approved by the Quality Improvement Executive Committee; as such, the authors utilized deidentified data from participant responses and did not perform informed consent, provide participants with monetary compensation, or register this program evaluation as a clinical trial.

## Results

A total of 130 Veterans participated in the virtual RWC programing across 14 cohorts from June 2020 to February 2022, with only 9 dropouts corresponding to a 93% completion rate. The mission of the Resilience & Wellness Center is to reduce suicide risk factors. Although a mental health diagnosis or history of suicidal behavior is not required to participate, following the authors' in-person approach^3^ to ascertain if the program interventions were specifically effective for those most at risk of suicide, the authors examined participants with history of suicidal ideation and attempt from those without separately.

**FIG. 1.**
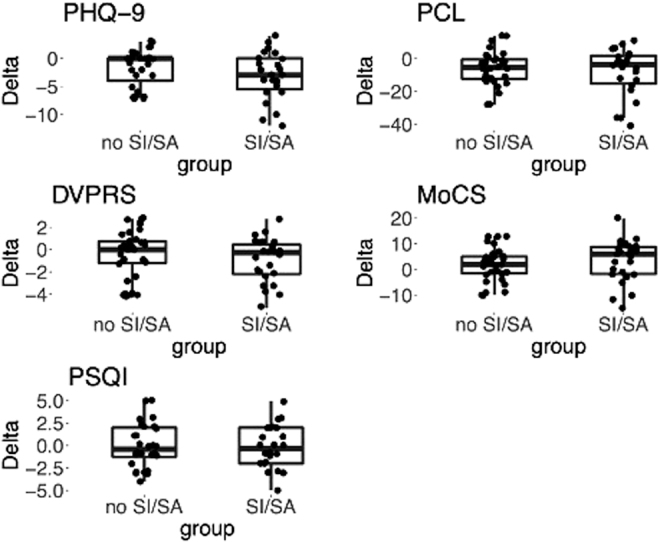
Program outcomes shown by group for no SI/SA and attempters. Boxplots show intraindividual differences in score [delta = (post-pre)] values for depressive symptoms (assessed by Patient Health Questionnaire-9), anxiety and stress symptoms (assessed by PTSD Checklist, Measures of Current Status), pain (assessed by Defense Veteran Pain Rating Scale), and sleep (assessed by Pittsburgh Sleep Quality Index). PTSD, post-traumatic stress disorder; SA, suicidal attempt; SI, suicidal ideation.

In particular, the authors sought to understand if Veterans who *were* at higher risk would show more significant improvements than those who did not. Of the 121 Veterans who completed the program, 46 (38%) had prior history of suicidal ideation (SI) or suicidal attempt (SA) with these at-risk Veterans being on the average 4.6 years younger than those with no history of suicidality (no SI/SA, see participant diagnostics and demographics details in Table 1). Assessments of the virtual RWC program outcomes for both mental and general health factors are reported in Figure 1 and Table 2.

**Table 1.** Demographic Data Representing 121 Participants Completing the Program

	Total* n* = 121	No SI/SA* n* = 75 (62%)	SI/SA* n* = 46 (38%)	*p*
Age (years)	55.03 ± 13.49	56.80 ± 13.22	52.15 ± 13.57	0.0656
Sex (female)	47 (39%)	26 (35%)	21 (46%)	0.3118
Ethnicity (Hispanic/Latino)^a^	40 (33%)	24 (32%)	16 (35%)	0.988
Race^b^				
Black/African American	72 (60%)	44 (59%)	28 (61%)	0.9446
White	36 (30%)	21 (28%)	15 (33%)	
American Indian/Alaskan	1 (1%)	1 (1%)	0 (0%)	
Asian	2 (2%)	1 (1%)	1 (2%)	
Native Hawaiian/Pacific Islander	1 (1%)	1 (1%)	0 (0%)	
Depression (yes)	109 (90%)	66 (88%)	43 (93%)	0.5323
PTSD (yes)	97 (80%)	55 (73%)	42 (91%)	0.0299
Substance use (yes)	52 (43%)	26 (35%)	26 (57%)	0.0301
Military sexual trauma (yes)^c^	31 (26%)	15 (20%)	16 (35%)	0.1208
Serious mental illness (yes)^d^	81 (67%)	46 (61%)	35 (76%)	0.0968

Significant differences observed only for PTSD and substance use status between No SI/SA and SI/SA group.

^a^ 2 No SI/SA reported unknown for ethnicity, test excluded unknowns.

^b^ For No SI/SA, 1 was missing for race, 6 declined to answer, 1 Native Hawaiian/Pacific Islander, 1 American Indian/Alaska Native, and 1 Asian; for SI/SA, 1 Asian, 1 declined to answer, and 1 reported unknown, statistical tests performed on Black/African and White only.

^c^ 1 No SI/SA was missing for military sexual trauma status.

^d^ 1 SI/SA was missing for serious mental illness.

SA, suicidal attempt; SI, suicidal ideation; PTSD, post-traumatic stress disorder.

**Table 2.** Program Outcomes by Suicidal Status

Outcomes	No SI/SA			SI/SA		
	*N*	Mean ± SD (adjusted* p*)	Cohen's d	*N*	Mean ± SD (adjusted* p*)	Cohen's d
Patient Health Questionnaire-9	27	−1.70 ± 3.30 (0.0063)	−0.52	23	−3.09 ± 4.34 (0.0025)	−0.71
PTSD Checklist	31	−5.70 ± 10.38 (0.0038)	−0.55	26	−8.86 ± 15.58 (0.0038)	−0.57
Defense Veteran Pain Rating Scale	34	−0.38 ± 2.16 (0.1599)	−0.17	26	−0.86 ± 1.94 (0.0337)	−0.44
Measures of Current Status	33	1.62 ± 6.45 (0.0793)	0.25	26	3.51 ± 8.01 (0.0348)	0.44
Pittsburgh Sleep Quality Index	28	−0.05 ± 2.42 (0.4589)	−0.02	24	−0.19 ± 2.39 (0.4589)	−0.08

Outcome measures with pre- vs. postdifferences are shown as mean delta scores by group. *p*-Values from one-sided *t* test were reported together with adjusted *p*-values using Benjamini–Hochberg method and effect size measured by Cohen's *d*.

These include data from 60 Veterans (49.5%) who completed both pre- and postassessment that were separated into two groups with/without prior history of suicide ideation and/or attempt (i.e., SI/SA and No SI/SA, respectively). Significant reduction in depression (through PHQ9) and PTSD (through PCL) in pre–post symptoms was observed for both groups with moderate effect sizes measured by Cohen's *d* (*d* > 0.5, Fig. 1 and Table 2). Significant reduction in pain symptoms (through DVPRS) for Veterans with suicide ideation and/or attempt history, as well as significant improvement in stress coping skills among this group (through MoCS) were observed with small effect sizes (DVPRS *d* = −0.44, and MoCS *d* = 0.44, Table 2).

No improvements in sleep quality were observed across both groups after RWC program completion (Table 2). Also, feedback on the experience of Veteran participants was evaluated to determine perceptions of the program and their experience. Responses are on a 5-point Likert scale ranging from “strongly disagree” to “strongly agree.” Veterans responded overwhelmingly favorably (Supplementary Fig. S1), noting that the telehealth CIH programming was easy to follow and understand, that the providers were helpful and that they were satisfied with the services received.

## Discussion

This program evaluation had a number of limitations, including small sample size, use of self-report validated clinical instruments for outcome assessments rather than clinician-administered, poor compliance with completion of pre–post assessments due to challenges by participants to access or ability to interface with the online Qualtrics XM software (e.g., Veterans with cognitive deficits), and lack of a control group for determination of treatment efficacy typical for a randomized clinical trial (RCT) framework. However, this is a quality improvement program evaluation with compelling results, motivating investigations of treatment efficacy in future RCT studies.

Specifically, the data showed that the delivery of multimodal CIH interventions through a virtual platform is highly promising, with participating Veterans reporting statistically significant reduction in depressive and PTSD symptoms over the course of the 4-week RWC programing. Remarkably, participation in the virtual multimodal CIH interventions also led to a decrease in pain and an increase in stress management skills in at-risk Veterans with prior history of suicide ideation and/or attempt.

The virtual mode of delivery of CIH interventions leverages the Department of Veterans Affairs existing infrastructure in internet-based telehealth platforms to increase access to care for Veterans. In 2018, the VA rolled out VVC as part of the “Anywhere to Anywhere Initiative,” which enabled clinicians to provide care nationwide, rather than being restricted by state lines, which led to almost 15% of all Veterans nationwide receiving care through telehealth that year, resulting in greater clinician encounters and reduction in missed appointments.^10^

The existing VA telehealth infrastructure was key in the authors' ability to pivot the RWC CIH interventions to a virtual mode of delivery in response to the COVID-19 pandemic. The transition to telehealth delivery also had unanticipated benefits, such as improving engagement in treatment for those with debilitating mental health issues, for whom attending in-person appointments was a frequent obstacle to care. Telehealth was also found to mitigate some of the lasting impacts of COVID, such as social isolation,^11,12^ a known risk factor for suicide.

To the authors' knowledge, this is the first telehealth CIH modality that includes at-risk suicidal Veterans in a targeted manner using virtual delivery of an immersive multimodal CIH intervention programing. While various CIH interventions are available and accessible to Veterans such as programs that focus on pain management through mindfulness meditation or tele-yoga,^13,14^ these are based on a single CIH treatment modality and do not specifically target at-risk suicidal Veterans. In addition, although these telehealth CIH interventions show early promise, data on effectiveness is lacking. Findings from this program evaluation is the first, showing that in line with the authors' in-person multimodal CIH intervention targeting at-risk Veterans, the virtual delivery appears to also be significantly effective in reducing mental health symptoms (i.e., depression and PTSD).

Remarkably, among specifically those Veterans with prior history of suicide ideation and/or attempt, the virtual delivery also resulted in reduction in symptoms of chronic pain and improved stress management skills that are key risk and protective factors in suicide risk and prevention. Taken together, virtual delivery of CIH interventions holds great promise in improving the health and wellness of suicidal Veterans, providing a stepped-care model for broadening suicide prevention efforts to at-risk populations nationwide.

## Supplementary Material

Supplemental data

Supplemental data
