# Supplementary material for: Delivery of Telehealth Complementary and Integrated Health Interventions Improves Mental Health and Overall Wellness to Broaden Veterans' Access to Care
Source: J Integr Complement Med. 2023 Feb 8;29(2):127–30. doi: 10.1089/jicm.2022.0614 (PMC10133970; doi:10.1089/jicm.2022.0614)
Supplement: Supplemental data [file Suppl_FigS1.docx]

| 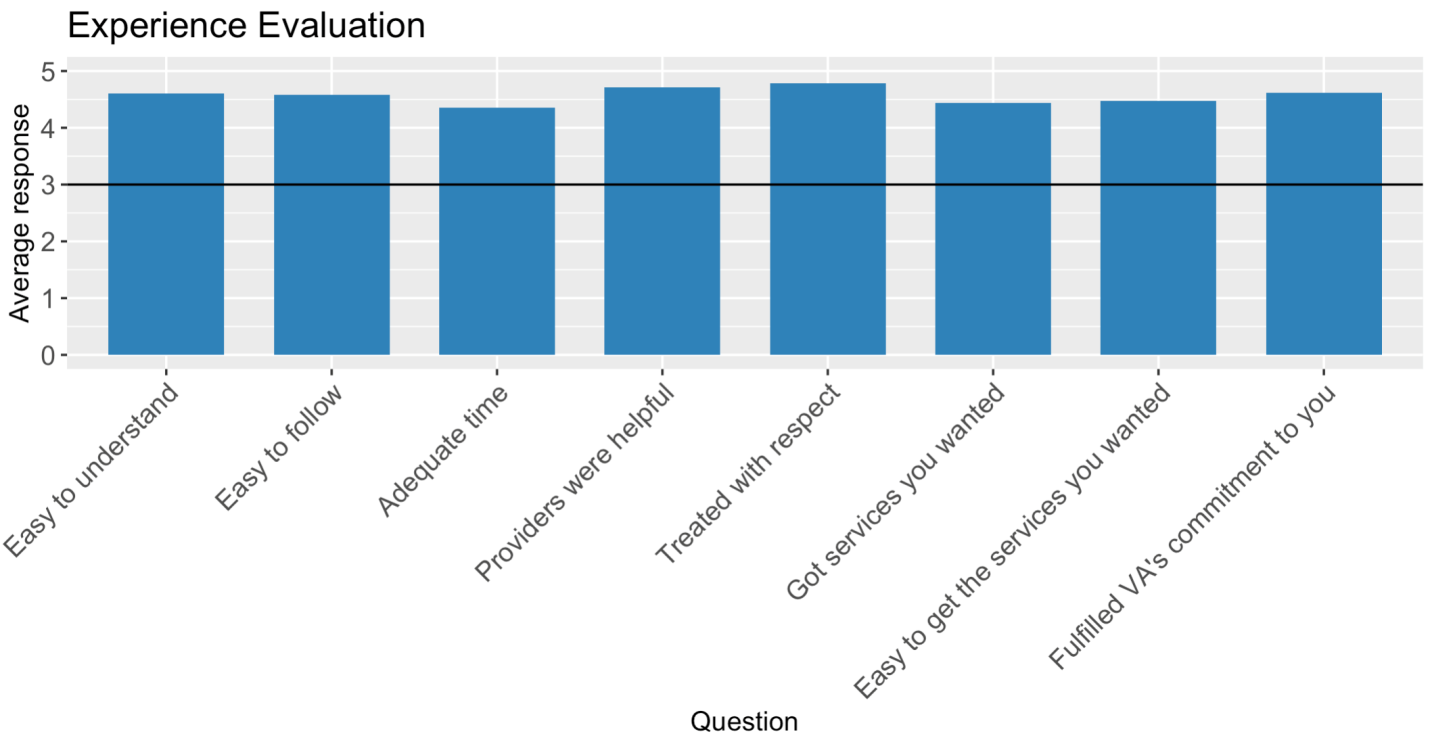 |
| --- |
| **Figure S1**: Program experience evaluation for total of 55 participants reported. More than 87% of participants either agreed or strongly agreed for all 8 criteria shown above. |
